# Revolutionizing plant biology: multiple ways of genome engineering by CRISPR/Cas

**DOI:** 10.1186/s13007-016-0103-0

**Published:** 2016-01-28

**Authors:** Simon Schiml, Holger Puchta

**Affiliations:** Botanical Institute II, Karlsruhe Institute of Technology, POB 6980, 76049 Karlsruhe, Germany

**Keywords:** Gene technology, Double-strand break repair, Synthetic nucleases, Targeted mutagenesis, Gene targeting

## Abstract

The precise manipulation of plant genomes relies on the induction of DNA double-strand breaks by site-specific nucleases to initiate DNA repair reactions that are either based on non-homologous end joining (NHEJ) or homologous recombination (HR). Recently, the CRISPR/Cas system emerged as the most important tool for genome engineering due to its simple structure and its applicability to a wide range of organisms. Here, we review the current status of its various applications in plants, where it is used for the successful generation of stable mutations in a steadily growing number of species through NHEJ. Furthermore, tremendous progress in plant genome engineering by HR was obtained by the setup of replicon mediated and *in planta* gene targeting techniques. Finally, other complex approaches that rely on the induction of more than one DNA lesion at a time such as paired nickases to avoid off-site effects or controlled genomic deletions are beginning to be applied routinely.

## Background

Although with the rise of the CRISPR/Cas (clustered regularly interspaced short palindromic repeats and CRISPR-associated) technology double-strand break (DSB)-induced genome engineering moved into the centre of scientific interest in the last 2 years, the basic principles behind this approach were well known for decades. Previous experiments demonstrated that by induction of a unique DSB in plant genomes using a highly specific endonuclease, different types of genome manipulations could be achieved. On one hand, the frequency of gene targeting (GT), the precise integration of a T-DNA via HR with sequences identical to the genomic locus, can be increased by several orders of magnitude [1]. On the other hand, functional open reading frames can be destroyed by imprecise NHEJ [2]. Interestingly, even in the absence of homologies, integration of a T-DNA into a genomic locus by NHEJ can be enhanced by DSB induction [2]. Depending on the location, the induction of two DSBs can result in deletions [3] or reciprocal exchanges between chromosome arms [4].

For many years, these experiments were performed using mega nucleases such as I-*Sce*I [5, 6]. However, this method required the introduction of the respective target sequence into the plant genome prior to the genome engineering experiment itself. Therefore, targeting endogenous loci is excluded, as engineering the protein for new target sequences is strongly limited [7, 8]. A major improvement was marked by the introduction of zinc finger nucleases (ZFNs) [9, 10]: the DNA cleavage domain from the restriction enzyme *Fok*I was fused to the highly variable DNA binding domain (DBD) of a class of zinc finger transcription factors. By combining different zinc fingers in the DBD, different target sites in the DNA are recognized and are cleaved by the nuclease. Unfortunately there are drawbacks to this technique as not all combinations of zinc fingers function well, therefore every new nuclease has to be tested extensively. Furthermore, cloning of a new nuclease is quite time consuming. However, ZFNs are still in use today primarily because there are no open questions regarding their status as intellectual property. The discovery and molecular analysis of transcription activator-like effectors (TALEs) from the plant pathogen species *Xanthomonas* led to the third important class of engineered nucleases [11]. The TALE DBD consists of numerous repeats, varying only in two amino acid residues. In 2009, it was shown that each of the repeating sequences is able to recognize and bind exactly one nucleotide on the DNA [12, 13]. An engineered TALE nuclease was created by fusing the DBD once again to *Fok*I, in these experiments, cloning of a new nuclease was facilitated by adapting the GoldenGate cloning method [14].

**Fig. 1:**
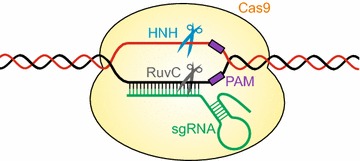
Schematic representation of the Cas9 cleavage mechanism. The Cas9/sgRNA complex recognizes and binds the complementary sequence next to the PAM, which is highly specific for each Cas9 from different bacterial species. The RuvC domain and HNH motif of Cas9 cleave the two DNA strands 3 bp upstream of the PAM

The most recent, yet already the most important type of programmable nucleases utilizes the CRISPR/Cas system. It was originally discovered in the 1980s as a distinctive genomic locus in *E. coli* [15, 16] and was later characterized to serve as an adaptive immune system in many bacteria and archaea [17]. However, the molecular mechanism of the CRISPR/Cas system from *Streptococcus pyogenes* was not deciphered until 2012 [18]: foreign plasmid or viral DNA entering the bacterial cells are degraded by a single protein, the nuclease Cas9. The target specificity is governed by a short so-called CRISPR-RNA (crRNA), which is encoded in the CRISPR-locus and which is complementary to the invading DNA so that it can bind directly to the foreign DNA using a stretch of 20 nts. An additional short sequence motif next to the target sequence, termed protospacer adjacent motif (PAM), is needed for the correct recognition of the target site. A second short RNA, the trans-activating CRISPR-RNA (tracrRNA), binds to the crRNA, and a stable complex is formed with Cas9. The foreign DNA is then cleaved by two nuclease domains of Cas9. Furthermore, it was shown that the two RNAs can also be fused together to form a so-called single-guide RNA (sgRNA, Fig. 1). At the beginning of 2013, the groups of Feng Zhang at MIT and George Church at Harvard demonstrated that Cas9 can be used for genome engineering in human cell cultures, proving that it also works in eukaryotic systems [19, 20]. Since then, the CRISPR/Cas system has made unprecedented success as a tool for genome engineering due to its ease in cloning new sequence-specific nucleases and the fact that it works in almost any organism. Here, we review the major technical advances that have been made with Cas9 in plants.

## Creating heritable mutations with RNA-guided Cas9 in plants

In August 2013, three studies were published in the same issue of nature biotechnology showing that Cas9 also works in plant cells. Scientists from the Chinese Academy of Sciences in Beijing demonstrated the disruption of endogenous genes in rice and wheat protoplasts as well as in rice calli [21] while researchers from Harvard reported cleavage activity of Cas9 in protoplasts of *Arabidopsis thaliana* and *Nicotiana benthamiana* [22]. Finally, the Sainsbury Lab in the UK used *N. benthamiana* leaf tissue for agroinfiltration, demonstrating Cas9-mediated disruption of the *PDS* gene (phytoene desaturase) [23].

However, stable inheritance of Cas9-mediated mutations in natural genes was not reported until 2014, when Feng et al. used the human codon-optimized Cas9 (hCas9) from F. Zhang [19] under the control of two CaMV 35S promoters and 12 different sgRNAs driven by the AtU6-26 promoter to target endogenous sites in *A. thaliana* [24]. U6 promoters control transcription by RNA polymerase III, which is specific for the production of short non-coding RNAs. The Cas9-harbouring constructs were stably integrated through *Agrobacterium tumefaciens*. T1 analyses revealed high mutation frequencies ranging from 30 % up to 92 % with 1-bp insertions being the dominant type of mutation. Investigation of T2 and T3 generations revealed Mendelian segregation and stable inheritance of mutations together with the rise of new somatic mutations, which are a result of ongoing DSB induction by the presence of the nuclease transgene. By using an interrupted GUS reporter construct [25], the ability of Cas9 to induce HR was also shown. By whole genome sequencing and Sanger sequencing of potential off-target sites, it was also demonstrated that the system is highly specific in plants as no significant off-target activity was detected. The same group also reported the generation of mutants for a set of genes in rice [26]. By transforming calli with subsequent regeneration of transgenic T0 plants, Cas9 activity could be observed early in plant development, leading to a high number of mutated plants. Additionally, heredity of some mutations to the T1 generation was demonstrated but not analysed in detail due to a high number of secondary mutations that created chimeric plants.

Soon after the reports mentioned above, the use of a distinct construct in *A. thaliana* was reported [27]. A codon optimized version of Cas9 is controlled by a plant Ubiquitin promoter, the sgRNA is expressed by the AtU6-26 promoter and both elements are located on the same T-DNA. Following stable transformation, homozygous mutations were induced at a frequency of up to 70 % in two endogenous marker genes of *A. thaliana* and in addition, inheritance of mutations into T2 and T3 generations as well as their segregation in a Mendelian fashion was demonstrated. Mutation patterns were analysed by next generation sequencing, which again exhibited small insertions of 1-bp to be the most dominant form of alteration. Furthermore, the first application of the Cas9-D10A nickase variant to plants was reported here. By inducing a point mutation to Cas9 that inactivates one of the nuclease domains, the nuclease is converted to a single-strand break (SSB) inducing nickase. NGS did not reveal a mutagenic potential of the nickase. However, when the constructs were applied to the HR reporter plant lines DGU.US and IU.GUS [28], the nickase was able to induce HR at least as efficiently as the Cas9 nuclease, rendering it a promising tool for HR-based genome engineering approaches.

Zhou et al. presented a study on the application of RNA-guided Cas9 in rice [29]. Here, a rice codon-optimized Cas9 was put under control of a maize ubiquitin promoter. Notably, two different sgRNAs, that have successfully been used in the mammalian system before (one with 48 nt tracr-tail [19] and one with 85 nt tracr tail [20]), as well as a dual guide system (dgRNA) with separate crRNA and tracrRNA, were used all under the control of a rice U6 promoter. When stably transformed to rice plants, the 48 nt sgRNA did not induce any detectable mutations, while the induction by the dgRNA was very low in the T0 generation (2 out of 16 transformants with a mono-allelic mutation). One might speculate, that these RNA constructs are not able to form a stable RNA-Cas9 complex under plant growth conditions. However, the 85 nt sgRNA did induce NHEJ events in the transformed generation with efficiencies ranging from 20 % up to 100 % of a small number of transformants. All of the mutations observed were already bi-allelic and the two mutated alleles showed Mendelian segregation in T1 and in transgene-free T2 plants. Furthermore, the induction of large deletions of up to 245 kb was observed in transgenic plants when two sgRNAs were applied, although inheritance was not investigated in this approach.

The van Eck group at the Cold Spring Harbor Laboratory was the first to demonstrate Cas9-mediated heritable mutations in tomato [30]. The hCas9 driven by the 35S promoter was combined with two sgRNAs under AtU6 control, intending to create a specific deletion in the *SlAGO7* gene for easy detection of mutations. Almost half of all transformants exhibited the recessive *wiry2*-*1* growth phenotype [31], indicating homozygous mutations early in development. Sanger sequencing confirmed the presence of respective mutations. However, in addition to the expected deletion, larger deletions that included the target size were observed, as well as small mutations affecting one or both of the target sequences. To show inheritance of mutations, wild-type flowers were pollinated by the mutant plants and the offspring were analysed. Heterozygous plants without the Cas9 transgene but with one wild-type and one mutant allele could be identified, thus confirming heredity of the mutations.

The group of Qi-Jun Chen from Beijing applied their RNA-guided Cas9 to *A. thaliana* and maize [32]. Using a set of different constructs (maize codon-optimised zCas9 or hCas9, Ubiquitin or 2 × 35S promoter, AtU6-26, OsU3 or TaU3 promoter for the sgRNA), targeted mutagenesis was demonstrated in both maize protoplasts and transgenic plants, with the combination of zCas9 and TaU3p exhibiting the highest efficiency. By using different Pol III promoters, it was also possible to assemble up to four different sgRNA expression cassettes on one vector for multiplex genome engineering. Heritability of mutations was confirmed in *A. thaliana* by checking for transgene-free T2 plants via PCR and subsequent sequencing of the Cas9 target sites. At the moment, the number of reports of new stably mutated plant species using CRISPR/Cas is rapidly growing [33, 34].

## Harnessing different Cas9 orthologues

A major step towards more complex applications of the CRISPR/Cas system was made by the adaption of additional Cas9 nucleases to genome engineering. The most widely used Cas9 nuclease originates from *Streptococcus pyogenes*. However, it was shown in mammalian cell culture that Cas9 orthologues from other species are also applicable for targeting unique genomic sites [35, 36]. Recently, it was shown that nucleases from *Streptococcus thermophilus* and *Staphylococcus aureus* also work efficiently in *A. thaliana* [37]. Stably transformed constructs contained a Cas9 expression system together with the species-specific sgRNA with distinct PAM-specificities. It was demonstrated that both nucleases led to highly efficient mutagenesis. For *S. aureus,* Cas9 targeting a specific PAM (‘NNGGGT’) increased the mutation frequency to almost 90 %, with the most dominant form of mutations being deletions. Mutations in the *ADH1* locus were shown to be heritable for both nucleases. Furthermore, it was demonstrated that cross interferences between Cas9 and sgRNA from different species do not occur. These findings provide the basis for more complex approaches, enabling the simultaneous control of different enzymatic activities in a single plant cell [38].

## Inducing genomic change during plant and organ development

Although the use of constitutive promoters exhibits high frequency mutagenesis for Cas9, other promoters are also desirable. This offers the possibility to achieve stable mutations more quickly, especially for plant species with long generation times. Additionally, conditional knockout or the mutagenesis of specific genes in unique organs is rendered possible by the use of respective promoters.

Together with scientists from Korea, George Coupland showed high-frequency mutagenesis in *A. thaliana* [39]. In their construct, instead of a constitutive promoter, the hCas9 was controlled by the *INCURVATA2* promoter, which is highly active in meristematic tissue. For the sgRNA, the AtU6-26 promoter was used. Mutagenesis frequencies for three endogenous targets ranged from 10 % to ~85 %, and T2 and T3 analysis revealed segregation of certain mutations as expected along with the induction of new mutations in the respective generation.

Qi-Jun Chen’s group from Beijing reported a similar experiment [40]: the zCas9 was put under control of the promoter from the egg cell-specific *EC1.2* of *A. thaliana* to increase heritability by inducing mutations in egg cells. Together with respective sgRNAs, T1 double and triple mutant *Arabidopsis* plants could be obtained that also segregated in the T2 generation. Furthermore, different combinations out of eight promoters and two terminators were analysed for their mutagenesis efficiency. The highest number of T1 triple mutants (17 %) was observed using a combination of the *EC1.2* enhancer and *EC1.1* promoter together with the *rbcS E9* terminator. Although this approach does not seem to significantly increase heritability of mutations compared to using a somatic expression system, having the ability to control mutagenesis through different developmental stages can be of great importance for studying the function of certain genes.

## Off-site effects and how to avoid them

A major concern when using an RNA-guided Cas9 is off-site activity. Although extensive studies that address this issue have been performed in the bacterial and mammalian system, the exact extent of off-site activity is still not completely clear. For plants, there is little data currently available that addresses off-site activity. Sequencing of bioinformatically identified putative off-target sites showed no detectable events in *A. thaliana*, *N. benthamiana*, wheat, rice and sweet orange [21–24, 29, 41, 42]. Whole-genome sequencing of mutated *A. thaliana* plants also resulted in no off-target events [24]. In contrast to these results, a study using Cas9 in rice found a putative off-target site to be mutated in 1.6 % of the investigated plants, although this was still five times less frequent than the on-target site [43]. A study covering the application of Cas9 to soybean reported an off-target frequency of 13 % [44]. It has to be noted, however, that in this study, two paralogues of *DDM1* were targeted and therefore the high sequence similarity is likely to cause the high amount of off-target activity.

**Fig. 2:**
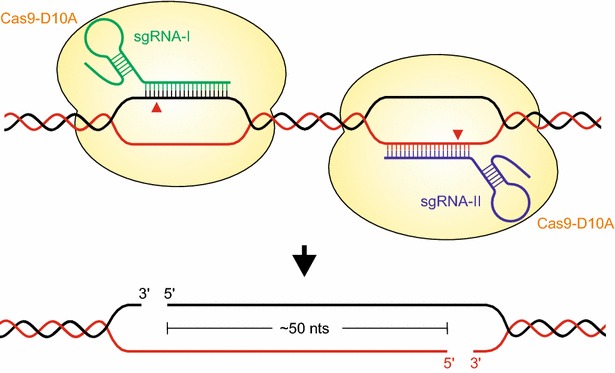
Cas9 paired nickases approach. By using 2 sgRNAs, the D10A nickase variant can be guided to the two opposite DNA strands at adjacent positions. The resulting BSD exhibits long single-stranded 5′-overhangs

A solution to off-target activity proposed by experiments in human cell culture is the use of two Cas9 nickases [45]. By introducing a point mutation (D10A) into one of the nuclease domains of Cas9, the enzyme is converted into a SSB inducing nickase. The nickase can then be guided to two adjacent positions in the genome by two distinct sgRNAs resulting in SSBs on each of the two DNA strands (Fig. 2). The result is a DSB that can also lead to NHEJ-mediated mutagenesis. Hence, the specificity is increased, as independent off-site binding of the sgRNAs does not lead to mutations. It has already been demonstrated that this approach is also applicable to plants. By adding a second sgRNA to a Cas9 construct and subsequent stable transformation into *A. thaliana*, a mutagenesis rate comparable to that of the Cas9 single nuclease was achieved [46]. Notably, the mutation pattern shifted from small insertions for the nuclease to larger deletions being the most dominant mutagenesis outcome. Again, mutations were proven to be heritable by demonstrating the presence of mutations in transgene-free T2 plants.

## Utilizing homologous recombination

**Fig. 3:**
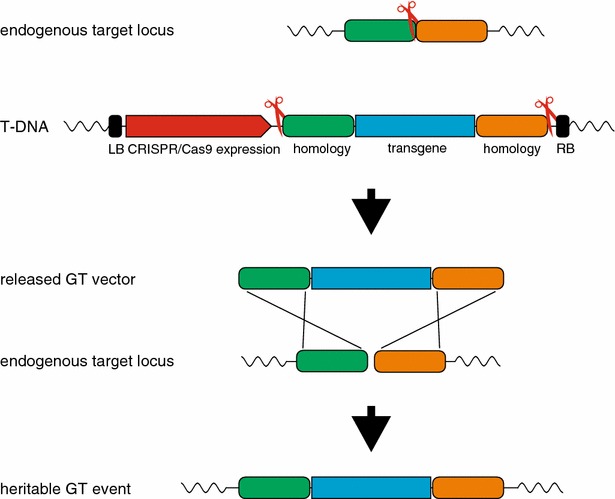
Overview of the Cas9-mediated *in planta* GT system. The nuclease and the DNA donor sequence are located on one T-DNA that is stably transformed into the plant. The nuclease induces two DSBs that release the donor intermediate and a third DSB that activates the target locus for HR. The donor sequence integrates into the target locus by using the flanking homologous regions

Site-specific integration of transgenes or precise genome alterations (referred to hereafter as gene targeting, GT) have always been major challenges in plant genome engineering. This is because NHEJ is by far the preferred mechanism to repair DNA breaks in somatic plant tissue. However, using the different classes of engineered nucleases, a wide variety of successful GT experiments have been performed [47]. A major step was taken with the development of the I-*Sce*I-based *in planta* GT system [48], which allowed for GT rates of more than 1 % in *A. thaliana* without having to rely on high transformation rates. Using Cas9, it was possible to improve this technique even further [46]: the number of T-DNAs needed for the system to work was reduced from three (donor sequence, artificial I-*Sce*I target sequence and I-*Sce*I expression system) to only one, harbouring both the donor sequence and the Cas9/sgRNA expression cassettes. Furthermore, the system’s target flexibility allowed for the targeting of an endogenous locus in the *Arabidopsis* genome. The precise site-specific integration of the donor sequence into this locus and the possible inheritance of this manipulation into the next generation were demonstrated by using the experimental setup shown in Fig. 3.

**Fig. 4:**
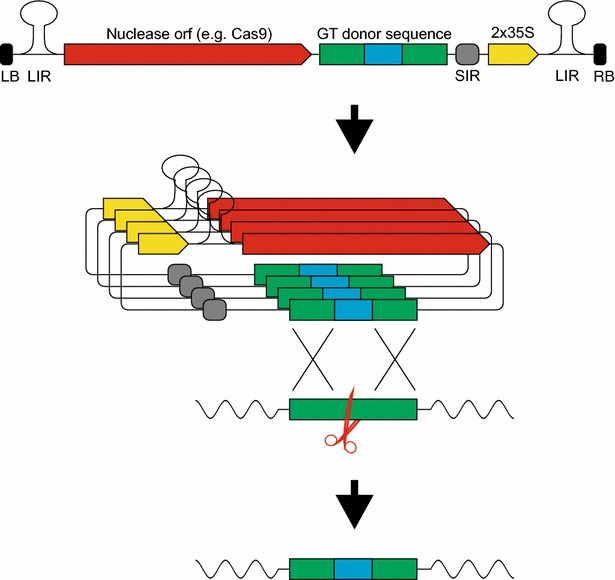
Replicon-mediated GT. The replicon is released from the T-DNA, circularises at the LIRs and undergoes rolling circle replication. This leads to the promoter being positioned upstream of the nuclease ORF. Upon DSB-induction in the target locus, the integration of the donor sequence can be achieved by HR

In an innovative approach, the group of Dan Voytas combined Cas9-mediated mutagenesis and GT with geminivirus-based replicons [49]. *Arabidopsis* plants were stably transformed with a specific T-DNA flanked by the viral large intergenic regions (LIRs). Upon co-transformation of the viral replication-initiation protein, replicational release, circularisation and rolling-circle replication of the replicon is initiated at the LIRs. The circularisation leads to two 35S promoters becoming correctly oriented in front of the desired gene, i.e., the nuclease ORF. With this strategy, NHEJ-mediated mutagenesis was demonstrated with ZFNs, TALENs and Cas9. Furthermore, it was shown that when a GT donor sequence was added to the replicon, ZFN-mediated GT could be achieved (Fig. 4). This strategy was also shown to be applicable using Cas9 in tomato [50].

Recently, scientists from DuPont Pioneer reported CRISPR/Cas-mediated GT in soybean [34]. Cas9-sgRNA constructs and a donor template were co-transformed into embryonic callus by particle bombardment. For two target sites on chromosome 4, the correct integration of the hygromycin resistance gene *HPT* was identified by PCR and confirmed by Southern blot in T0, and respective plants were regenerated. For one target in T1, three plants with the correct GT event and without any additional transgenes could be identified, confirming inheritance of the new allele. For the second target, only events with additional integration of either Cas9 at the target site or the donor template at random genomic sites were isolated. Notably, the amount of donor- and sgRNA-Cas9-DNA was not optimised to obtain true GT events. Furthermore, as this was a proof-of-principle study, only a small number of events was regenerated, and a true GT event for the second target is likely to be found when analysing a higher number of events.

## Multiplex genome engineering

The architecture of the CRISPR/Cas system with the constant Cas9 protein and the sgRNA-derived target specificity provides the opportunity to target multiple sites at once, as is the case in the natural bacterial system. However, we are only beginning to exploit this possibility, and successful imitation of the bacterial system with polycistronic crRNAs and tracrRNAs has not yet been reported for plants. Therefore, the most common approach by some groups to achieve multiplex sgRNA expression is to simply assemble numerous sgRNA expression systems, each with its own promoter [32, 46, 51]. However, this method is limited as constructs become very large with a Pol III promoter for every sgRNA. Xie et al. developed a clever solution to overcome this problem [52]: tRNA sequences were put in between the sgRNA sequences on the construct to create a single polycistronic gene. Two host-endogenous nucleases cleave the expressed RNA at the borders between tRNA and sgRNA creating individual sgRNAs. Simultaneous multiplex mutagenesis was demonstrated in rice protoplasts and transgenic plants for different numbers of sgRNAs (up to eight) in a tRNA-sgRNA array.

Scientists from KAUST in Saudi Arabia demonstrated the potential of plant viruses for multiplex genome engineering [53]. In their study, the Tobacco rattle virus (TRV) was used to deliver sgRNAs to transgenic *N. benthamiana* that stably overexpressed Cas9. The two TRV RNAs were introduced into the plants by leaf agroinfiltration with a mixture of two bacterium cultures, one with a plasmid for RNA1, one for RNA2. The latter contained the sgRNA expression system. After reconstitution of the TRV in the infiltrated tissue, a systemic infection throughout the plant leads to sgRNA expression in all tissues and therefore efficient mutagenesis. By mixing bacterial cultures with different RNA2 vectors, simultaneous mutagenesis of two loci was achieved, indicating the potential for multiplex genome engineering by TRV-mediated sgRNA delivery.

## Controlling transcription with Cas9

Targeted genome manipulations with RNA-guided Cas9 have not only been achieved by altering the genome itself but also by influencing transcription. By guiding a catalytically inactive dead (d) Cas9 to a promoter or coding region, transcription can be efficiently blocked [54]. This effect can be enhanced by fusing a repressor protein such as the KRAB domain to dCas9. Likewise, an activator such as VP64 can also be fused to targeted dCas9 to activate transcription of a specific gene [55].

Magdy Mahfouz’s group from KAUST was able to transfer this system to plants [56]. The C-terminus of the human codon-optimized dCas9 was fused to the EDLL domain [57] or to the TAL activation domain [58] to create artificial activators. A repressor was created by fusing the SRDX EAR motif [59]. Successful transcriptional activation or repression could be observed in infiltrated *N. benthamiana* leaves by measuring expression levels of a transient *GUS* gene or the endogenous *PDS*. Activation was highest when the activator was guided to the sense strand of the promoter near the transcriptional start site and both activator constructs performed at a comparable level. Repression of PDS was demonstrated for both dCas9 alone and for the dCas9: SRDX fusion construct and could be increased by guiding the complex to several target sites within the promoter and the first exon of the gene simultaneously.

## Conclusions and outlook

In the last 2 years, CRISPR/Cas has emerged as the most important tool for molecular biology due to its simplicity, versatility and efficiency. The immediate benefit for plant scientists is the possibility to rapidly create mutations in genes where no known T-DNA insertion or EMS mutant is available. Use of this method will therefore lead to a more complete understanding of gene function in plants. This approach can not only be applied to genes with unknown functions but also to genes for which we must revise our current knowledge due to the option to produce true knock-out mutants. For instance, scientists from UCSD recently demonstrated that a putative key player of auxin signalling in Arabidopsis (*ABP1*) does not possess the important function that was inferred from the analysis of plants obtained by more classical mutagenesis techniques [60]. However, NHEJ-mediated mutation is only a first, yet important step. Specific changes of single amino acids or integration of a larger piece of DNA in the plant genome can be achieved by using Cas9-based GT systems, while efficient multiplex systems will allow the complex rearrangement of chromosomes. Thus, as well as coming closer to developing synthetic plant genomes, we will be also able to obtain plants with a single engineered point mutation that cannot be discriminated from natural varieties. Such plants can even be obtained without the use of transgenic DNA [61], which will also help accelerate the acceptance of Cas9 mutagenized crop plants for agricultural use in the general public.

## References

[1] Puchta H, Dujon B, Hohn B (1996). Two different but related mechanisms are used in plants for the repair of genomic double-strand breaks by homologous recombination. Proc Natl Acad Sci USA.

[2] Salomon S, Puchta H (1998). Capture of genomic and T-DNA sequences during double-strand break repair in somatic plant cells. EMBO J.

[3] Siebert R, Puchta H (2002). Efficient repair of genomic double-strand breaks by homologous recombination between directly repeated sequences in the plant genome. Plant Cell.

[4] Pacher M, Schmidt-Puchta W, Puchta H (2007). Two unlinked double-strand breaks can induce reciprocal exchanges in plant genomes via homologous recombination and nonhomologous end joining. Genetics.

[5] Puchta H (2005). The repair of double-strand breaks in plants: mechanisms and consequences for genome evolution. J Exp Bot.

[6] Jacquier A, Dujon B (1985). An intron-encoded protein is active in a gene conversion process that spreads an intron into a mitochondrial gene. Cell.

[7] Steuer S, Pingoud V, Pingoud A, Wende W (2004). Chimeras of the homing endonuclease PI-SceI and the homologous Candida tropicalis intein: a study to explore the possibility of exchanging DNA-binding modules to obtain highly specific endonucleases with altered specificity. Chem Biochem.

[8] Gimble FS, Moure CM, Posey KL (2003). Assessing the plasticity of DNA target site recognition of the PI-SceI homing endonuclease using a bacterial two-hybrid selection system. J Mol Biol.

[9] Kim YG, Cha J, Chandrasegaran S (1996). Hybrid restriction enzymes: zinc finger fusions to Fok I cleavage domain. Proc Natl Acad Sci USA.

[10] Smith J, Bibikova M, Whitby FG, Reddy AR, Chandrasegaran S, Carroll D (2000). Requirements for double-strand cleavage by chimeric restriction enzymes with zinc finger DNA-recognition domains. Nucleic Acids Res.

[11] Bonas U, Stall RE, Staskawicz B (1989). Genetic and structural characterization of the avirulence gene avrBs3 from *Xanthomonas campestris* pv. vesicatoria. Mol Gen Genet.

[12] Boch J, Scholze H, Schornack S, Landgraf A, Hahn S, Kay S (2009). Breaking the code of DNA binding specificity of TAL-type III effectors. Science.

[13] Moscou MJ, Bogdanove AJ (2009). A simple cipher governs DNA recognition by TAL effectors. Science.

[14] Cermak T, Doyle EL, Christian M, Wang L, Zhang Y, Schmidt C (2011). Efficient design and assembly of custom TALEN and other TAL effector-based constructs for DNA targeting. Nucleic Acids Res.

[15] Stern MJ, Ames GF, Smith NH, Robinson EC, Higgins CF (1984). Repetitive extragenic palindromic sequences: a major component of the bacterial genome. Cell.

[16] Ishino Y, Shinagawa H, Makino K, Amemura M, Nakata A (1987). Nucleotide sequence of the iap gene, responsible for alkaline phosphatase isozyme conversion in *Escherichia coli*, and identification of the gene product. J Bacteriol.

[17] Wiedenheft B, Sternberg SH, Doudna JA (2012). RNA-guided genetic silencing systems in bacteria and archaea. Nature.

[18] Jinek M, Chylinski K, Fonfara I, Hauer M, Doudna JA, Charpentier E (2012). A programmable dual-RNA-guided DNA endonuclease in adaptive bacterial immunity. Science.

[19] Cong L, Ran FA, Cox D, Lin S, Barretto R, Habib N (2013). Multiplex genome engineering using CRISPR/Cas systems. Science.

[20] Mali P, Yang L, Esvelt KM, Aach J, Guell M, DiCarlo JE (2013). RNA-guided human genome engineering via Cas9. Science.

[21] Shan Q, Wang Y, Li J, Zhang Y, Chen K, Liang Z (2013). Targeted genome modification of crop plants using a CRISPR-Cas system. Nat Biotechnol.

[22] Li J, Norville JE, Aach J, McCormack M, Zhang D, Bush J (2013). Multiplex and homologous recombination-mediated genome editing in *Arabidopsis* and *Nicotiana benthamiana* using guide RNA and Cas9. Nat Biotechnol.

[23] Nekrasov V, Staskawicz B, Weigel D (2013). Jones, Jonathan D G, Kamoun S. Targeted mutagenesis in the model plant *Nicotiana benthamiana* using Cas9 RNA-guided endonuclease. Nat Biotechnol.

[24] Feng Z, Mao Y, Xu N, Zhang B, Wei P, Yang D (2014). Multigeneration analysis reveals the inheritance, specificity, and patterns of CRISPR/Cas-induced gene modifications in *Arabidopsis*. Proc Natl Acad Sci USA.

[25] Mao Y, Zhang H, Xu N, Zhang B, Gou F, Zhu J (2013). Application of the CRISPR-Cas system for efficient genome engineering in plants. Mol Plant.

[26] Zhang H, Zhang J, Wei P, Zhang B, Gou F, Feng Z (2014). The CRISPR/Cas9 system produces specific and homozygous targeted gene editing in rice in one generation. Plant Biotechnol J.

[27] Fauser F, Schiml S, Puchta H (2014). Both CRISPR/Cas-based nucleases and nickases can be used efficiently for genome engineering in *Arabidopsis**thaliana*. Plant J..

[28] Orel N, Kyryk A, Puchta H (2003). Different pathways of homologous recombination are used for the repair of double-strand breaks within tandemly arranged sequences in the plant genome. Plant J..

[29] Zhou H, Liu B, Weeks DP, Spalding MH, Yang B (2014). Large chromosomal deletions and heritable small genetic changes induced by CRISPR/Cas9 in rice. Nucleic Acids Res.

[30] Brooks C, Nekrasov V, Lippman ZB, van Eck J (2014). Efficient gene editing in tomato in the first generation using the clustered regularly interspaced short palindromic repeats/CRISPR-associated9 system. Plant Physiol.

[31] Yifhar T, Pekker I, Peled D, Friedlander G, Pistunov A, Sabban M (2012). Failure of the tomato trans-acting short interfering RNA program to regulate AUXIN RESPONSE FACTOR3 and ARF4 underlies the wiry leaf syndrome. Plant Cell..

[32] Xing H, Dong L, Wang Z, Zhang H, Han C, Liu B (2014). A CRISPR/Cas9 toolkit for multiplex genome editing in plants. BMC Plant Biol.

[33] Schaeffer SM, Nakata PA (2015). CRISPR/Cas9-mediated genome editing and gene replacement in plants: transitioning from lab to field. Plant Sci.

[34] Li Z, Liu Z, Xing A, Moon BP, Koellhoffer JP, Huang L (2015). Cas9-guide RNA directed genome editing in soybean. Plant Physiol.

[35] Ran FA, Cong L, Yan WX, Scott DA, Gootenberg JS, Kriz AJ (2015). In vivo genome editing using *Staphylococcus aureus* Cas9. Nature.

[36] Esvelt KM, Mali P, Braff JL, Moosburner M, Yaung SJ, Church GM (2013). Orthogonal Cas9 proteins for RNA-guided gene regulation and editing. Nat Methods.

[37] Steinert J, Schiml S, Fauser F, Puchta H. Highly efficient heritable plant genome engineering using Cas9 orthologues from *Streptococcus thermophilus* and *Staphylococcus aureus*. Plant J. 2015. 10.1111/tpj.13078 26576927

[38] Puchta H. Using CRISPR/Cas in three dimensions. Towards synthetic plant genomes, transcriptomes and epigenomes. Plant J. 2015. 10.1111/tpj.13100 26677816

[39] Hyun Y, Kim J, Cho SW, Choi Y, Kim J, Coupland G (2015). Site-directed mutagenesis in *Arabidopsis thaliana* using dividing tissue-targeted RGEN of the CRISPR/Cas system to generate heritable null alleles. Planta.

[40] Wang Z, Xing H, Dong L, Zhang H, Han C, Wang X (2015). Egg cell-specific promoter-controlled CRISPR/Cas9 efficiently generates homozygous mutants for multiple target genes in *Arabidopsis* in a single generation. Genome Biol.

[41] Jia H, Wang N (2014). Targeted genome editing of sweet orange using Cas9/sgRNA. PLoS One.

[42] Upadhyay SK, Kumar J, Alok A, Tuli R. RNA-guided genome editing for target gene mutations in wheat. G3 (Bethesda). 2013;3:2233–8. doi:10.1534/g3.113.008847. 10.1534/g3.113.008847 PMC3852385 24122057

[43] Xie K, Yang Y (2013). RNA-guided genome editing in plants using a CRISPR-Cas system. Mol Plant.

[44] Jacobs TB, LaFayette PR, Schmitz RJ, Parrott WA (2015). Targeted genome modifications in soybean with CRISPR/Cas9. BMC Biotechnol.

[45] Ran FA, Hsu PD, Lin C, Gootenberg JS, Konermann S, Trevino AE (2013). Double nicking by RNA-guided CRISPR Cas9 for enhanced genome editing specificity. Cell.

[46] Schiml S, Fauser F, Puchta H (2014). The CRISPR/Cas system can be used as nuclease for in planta gene targeting and as paired nickases for directed mutagenesis in *Arabidopsis* resulting in heritable progeny. Plant J..

[47] Puchta H, Fauser F (2013). Gene targeting in plants: 25 years later. Int J Dev Biol.

[48] Fauser F, Roth N, Pacher M, Ilg G, Sánchez-Fernández R, Biesgen C (2012). In planta gene targeting. Proc Natl Acad Sci USA.

[49] Baltes NJ, Gil-Humanes J, Cermak T, Atkins PA, Voytas DF (2014). DNA replicons for plant genome engineering. Plant Cell.

[50] Čermák T, Baltes NJ, Čegan R, Zhang Y, Voytas DF (2015). High-frequency, precise modification of the tomato genome. Genome Biol.

[51] Ma X, Zhang Q, Zhu Q, Liu W, Chen Y, Qiu R (2015). A Robust CRISPR/Cas9 system for convenient, high-efficiency multiplex genome editing in monocot and dicot plants. Mol Plant.

[52] Xie K, Minkenberg B, Yang Y (2015). Boosting CRISPR/Cas9 multiplex editing capability with the endogenous tRNA-processing system. Proc Natl Acad Sci USA.

[53] Ali Z, Abul-Faraj A, Li L, Ghosh N, Piatek M, Mahjoub A (2015). Efficient virus-mediated genome editing in plants using the CRISPR/Cas9 system. Mol Plant.

[54] Qi LS, Larson MH, Gilbert LA, Doudna JA, Weissman JS, Arkin AP (2013). Repurposing CRISPR as an RNA-guided platform for sequence-specific control of gene expression. Cell.

[55] Gilbert LA, Larson MH, Morsut L, Liu Z, Brar GA, Torres SE (2013). CRISPR-mediated modular RNA-guided regulation of transcription in eukaryotes. Cell.

[56] Piatek A, Ali Z, Baazim H, Li L, Abulfaraj A, Al-Shareef S (2015). RNA-guided transcriptional regulation in planta via synthetic dCas9-based transcription factors. Plant Biotechnol J.

[57] Tiwari SB, Belachew A, Ma SF, Young M, Ade J, Shen Y (2012). The EDLL motif: a potent plant transcriptional activation domain from AP2/ERF transcription factors. Plant J..

[58] Morbitzer R, Römer P, Boch J, Lahaye T (2010). Regulation of selected genome loci using de novo-engineered transcription activator-like effector (TALE)-type transcription factors. Proc Natl Acad Sci USA.

[59] Hiratsu K, Matsui K, Koyama T, Ohme-Takagi M (2003). Dominant repression of target genes by chimeric repressors that include the EAR motif, a repression domain, in *Arabidopsis*. Plant J..

[60] Gao Y, Zhang Y, Zhang D, Dai X, Estelle M, Zhao Y (2015). Auxin binding protein 1 (ABP1) is not required for either auxin signaling or *Arabidopsis* development. Proc Natl Acad Sci USA.

[61] Woo JW, Kim J, Kwon SI, Corvalán C, Cho SW, Kim H (2015). DNA-free genome editing in plants with preassembled CRISPR-Cas9 ribonucleoproteins. Nat Biotechnol.

